# Physician Well-Being, Burnout Symptoms, and Workplace Characteristics Among CARRA-Member Pediatric Rheumatologists in North America: Findings from a 2024 Cross-Sectional Survey

**DOI:** 10.3390/healthcare14213687

**Published:** 2026-10-31

**Authors:** Kathryn C. Cook, Aviya L. Levy, Lakshmi Nandini Moorthy, Arielle Hay, Daniel Glaser, Sarah Hoffmann, Farzana Nuruzzaman, Onengiya Harry, Deepika Singh, Sheetal S. Vora

**Affiliations:** 1 Akron Children’s Hospital, Akron, OH 44308, USA; 2 Rady Children’s Hospital, San Diego, CA 92123, USA; 3 Pediatrics, Vc-Health Sciences-Cchools, University of California San Diego, San Diego, CA 92093, USA; 4 Division of Pediatric Rheumatology, Rutgers-Robert Wood Johnson Medical School, New Brunswick, NJ 08901, USA; 5 Nicklaus Children’s Hospital Health System, Miami, FL 33155, USA; 6 Yale New Haven Children’s Hospital, New Haven, CT 06510, USA; 7 Children’s Hospital of Richmond at VCU, Richmond, VA 23219, USA; 8 Renaissance School of Medicine, Stony Brook University, Stony Brook, NY 11794, USA; 9 School of Medicine, Wake Forest University, Winston Salem, NC 27157, USA; 10 Valley Children’s Healthcare, Madera, CA 93636, USA; 11 Atrium Health Levine Children’s Hospital, Charlotte, NC 28203, USA

**Keywords:** pediatric rheumatology, physician burnout, physician well-being, occupational stress, workforce sustainability, work–life integration, electronic health records, organizational factors, workplace characteristics, healthcare workforce

## Abstract

**Background:** Physician burnout threatens clinician well-being, job satisfaction, retention, and access to care. Pediatric rheumatology faces documented workforce shortages, yet little specialty-specific information exists regarding physician well-being, burnout symptoms, and workplace characteristics. **Methods:** We conducted a cross-sectional descriptive survey of CARRA-registered pediatric rheumatologists in North America between January and March 2024. Participants completed the Mayo Clinic Well-Being Index (WBI), followed by the AMA Organizational Biopsy^®^ survey incorporating the Mini-Z 2.0 and demographic/workplace questions. Descriptive statistics summarized physician well-being, burnout symptoms, and workplace characteristics. Differences in WBI scores across response categories for two WBI anchor items assessing work–life integration and work meaningfulness were evaluated using ANOVA. AMA workplace data were available only in aggregate form and could not be linked to individual WBI responses. **Results:** Of 330 invited pediatric rheumatologists, 164 (49.7%) completed the WBI survey and 145 (88.4%) completed the redirected AMA survey. The mean WBI score was 2.7 (SD 1.9), and 36.0% scored ≥4. On the Mini-Z single-item measure, 48.3% of respondents endorsed at least one category consistent with burnout symptoms; by comparison, 64.0% endorsed the WBI item asking whether they had felt burned out from work during the past month. Frequently reported workplace challenges included documentation burden (56.6%), EHR-related frustration (59.3%), and after-hours EHR work (55.2%). Work–life integration was significantly associated with WBI scores (ANOVA *p* < 0.001), whereas meaningfulness of work was not (*p* = 0.26). **Conclusions:** Indicators of burnout symptoms and occupational distress were common among the responding pediatric rheumatologists. Frequently reported workplace challenges and the observed association between work–life integration and WBI scores identify potential areas for future organizational well-being initiatives.

## 1. Introduction

Physician burnout is an increasingly recognized challenge across healthcare because of its adverse effects on physician well-being, patient care, healthcare operations, and workforce sustainability [1]. In 2022, the U.S. Surgeon General identified health worker burnout as a critical threat to the healthcare workforce and highlighted organizational contributors including administrative burden, workforce shortages, and workflow inefficiencies [2]. Burnout is commonly conceptualized as a psychological syndrome arising from chronic workplace stress and is characterized by emotional exhaustion, depersonalization or cynicism, and a reduced sense of professional accomplishment [3]. Although burnout has been studied across multiple medical specialties, little is known about physician well-being, burnout symptoms, and workplace factors among pediatric rheumatologists. Given ongoing workforce shortages within the specialty, understanding physician well-being and burnout has become increasingly important [4].

The pediatric rheumatology workforce in the United States remains insufficient to meet projected national demand, leaving many regions with limited access to care [4]. Similar challenges have been reported in Canada, where workforce analyses have identified shortages of pediatric rheumatologists and substantial regional variation in access to care [5]. These workforce challenges underscore the importance of understanding factors that may affect the well-being and sustainability of the practicing workforce. Although workforce reports have described physician supply, workforce projections, and access-to-care challenges, less is known about burnout symptoms and workplace characteristics among practicing pediatric rheumatologists [4,5]. Concerns regarding physician well-being have also been documented among practicing rheumatologists more broadly. In the 2020 Canadian Rheumatology Association Workforce and Wellness Survey, approximately half of respondents reported burnout, highlighting the relevance of physician well-being to rheumatology workforce sustainability [6]. However, these findings do not fully characterize the well-being and workplace experiences of pediatric rheumatologists in North America. Specialty-specific data are needed to better describe occupational distress and commonly reported workplace challenges in this population.

Pediatric rheumatologists care for children with complex, chronic, and often lifelong conditions, requiring longitudinal relationships with patients and families, multidisciplinary coordination, and intensive medical decision-making. These responsibilities occur in the context of workforce shortages and competing clinical and academic demands. In our previous study, we reported high levels of burnout despite high resilience among pediatric rheumatology fellows, suggesting that well-being challenges warrant attention across the training and career continuum [7]. Whereas our prior study examined wellness, resilience, and burnout among pediatric rheumatology fellows, the present study focuses on practicing pediatric rheumatologists and describes workplace culture and organizational characteristics using the AMA Organizational Biopsy^®^ platform. This approach provides a broader description of physician well-being and workplace experiences within the established pediatric rheumatology workforce.

Organizational interventions such as workload redistribution, adequate support staff, team-based care, protected academic time, flexible scheduling, and electronic health record (EHR) workflow optimization have been proposed to reduce physician burnout and improve professional well-being [2,8]. Understanding the workplace challenges reported by pediatric rheumatologists may help inform future organizational assessment and the development and evaluation of specialty-relevant well-being initiatives.

The primary objective of this study was to describe physician well-being and burnout symptoms among CARRA-member pediatric rheumatologists in North America. Secondary objectives were to describe workplace characteristics measured through the AMA Organizational Biopsy^®^/Mini-Z 2.0 platform and to examine differences in Mayo Clinic Well-Being Index (WBI) scores across response categories for the two additional WBI anchor items assessing work meaningfulness and work–life integration.

## 2. Materials and Methods

### 2.1. Instrument Selection

Members of the Childhood Arthritis and Rheumatology Research Alliance (CARRA) Small Center Workgroup (SCW) identified physician wellness and burnout as important issues affecting the pediatric rheumatology workforce, and collaborated to select validated assessment tools and develop supplemental questions that addressed relevant practice-specific factors across diverse clinical settings. The SCW is a stakeholder committee within CARRA established to promote equitable participation of small pediatric rheumatology centers in collaborative research initiatives. Small centers are defined by CARRA as sites with three or fewer clinical full-time-equivalent (FTE) pediatric rheumatology physicians.

The use of two assessment platforms was intentional and reflected the distinct objectives of the study. The WBI was selected for assessment of individual physician distress and risk for adverse personal and professional outcomes, while the AMA Organizational Biopsy^®^ platform (American Medical Association, Chicago, IL, USA) was used to evaluate workplace culture, organizational factors, and practice characteristics that are vital for physician well-being. Together, these instruments provided complementary perspectives on physician wellness by evaluating both individual distress and organizational factors in the physician workplace.

#### 2.1.1. WBI

The WBI is a validated screening instrument designed to identify physician distress and reduced quality of life across different domains, including burnout, fatigue, depression, and mental and physical well-being [9,10]. This instrument was selected because of its established validity, brevity, and widespread use in assessing occupational distress among healthcare professionals [9,10]. The WBI was used to evaluate individual physician well-being and identify respondents at increased risk for adverse personal and professional consequences related to occupational distress. The version of the WBI used in this study includes seven scored dichotomous (yes/no) items that contribute to the total WBI score, as well as two additional response-scaled items (agree strongly/agree/neither agree nor disagree/disagree/strongly disagree) [9,10]. Higher WBI scores indicate greater occupational distress and increased risk of adverse personal and professional outcomes. Consistent with prior validation studies of the seven-item physician WBI, a score of ≥4 was used to identify physicians at elevated risk of adverse outcomes, including burnout, severe fatigue, reduced quality of life, suicidal ideation, and intention to leave practice. The primary WBI outcome was the validated seven-item physician WBI score (range 0–7). The two additional anchor questions assessing meaningfulness of work and work–life integration were analyzed separately and were not included in score calculation. The WBI was made available at no cost through an existing license at the lead author’s institution. The WBI typically requires less than 5 min to complete.

#### 2.1.2. Mini-Z

The Mini-Z 2.0 is a validated instrument used to assess physician burnout and workplace factors associated with stress and professional satisfaction, including workload, teamwork, autonomy, and electronic health record (EHR) burden [11,12]. This instrument has demonstrated acceptable psychometric properties and includes a burnout measure that correlates with established burnout assessments. The Mini-Z 2.0 requires approximately 2 to 3 min to complete.

#### 2.1.3. American Medical Association (AMA) Organizational Biopsy^®^ Platform

The AMA Organizational Biopsy^®^ is a standardized physician well-being assessment and benchmarking platform designed to help healthcare organizations measure professional well-being and identify workplace factors affecting clinicians. Through collaboration between CARRA and the AMA, the AMA provided access to the Mini-Z 2.0 instrument, assisted with survey development and data management, and facilitated collection of aggregate workplace culture and demographic information at no charge. The Organizational Biopsy^®^ was used with permission of the American Medical Association and is protected by copyright. CARRA and the AMA are separate organizations with no financial relationship or financial dependence on one another. Their collaboration was limited to the conduct of this survey and does not represent an ongoing organizational partnership.

#### 2.1.4. Demographic and Workplace Questions

A list of questions addressing demographic and organizational workflow in clinics was developed by the CARRA SCW with assistance from the AMA.

Additional items collected demographic and practice-related information, including commute characteristics, workplace culture, practice size, clinical responsibilities, outpatient clinical effort, physician on-call burden, academic activities, workplace scheduling, compensation, participation in professional activities, and other workforce-related domains. Because the primary objective of the present study was to evaluate physician well-being, burnout, work–life integration, meaningful work, and selected workplace culture characteristics, many workforce-related variables collected through the Organizational Biopsy^®^ were not included in the current analyses and may be the subject of future investigation. These supplemental survey items were developed through an iterative review process by the CARRA SCW in collaboration with the AMA. The supplemental demographic and practice-related questions required approximately 15–20 min to complete. Total survey completion time, including the WBI and Mini-Z 2.0 components, was approximately 20–30 min.

### 2.2. Subjects

Eligible participants were physicians who were registered CARRA members and identified pediatric rheumatology as their specialty in North America. Individuals were excluded if they were in training, including fellows and residents, physicians from other specialties, non-physician healthcare providers, patients, or caregiver members of CARRA. Eligible participants were identified through the CARRA membership directory after filtering for pediatric rheumatologists, and invitation emails containing links to the anonymous surveys were distributed to 330 physicians. The sampling frame was derived from the CARRA membership directory and included all physicians identified as practicing pediatric rheumatologists in North America who met the study eligibility criteria at the time of recruitment. Because the study sought to invite all eligible pediatric rheumatologists identified through the directory, the recruitment strategy represented an attempted census of the accessible eligible population rather than probability- or convenience-based sampling. The invitation email described the purpose of the study and provided information regarding participation and consent. Participation was voluntary, and participants provided informed consent before completing the anonymous survey. Nonrespondents received reminder emails every two weeks after the initial invitation, for a total of three reminders. No financial incentives were provided for participation. The study was reviewed and determined to be exempt by the Akron Children’s Hospital Institutional Review Board (Study #2022-137).

### 2.3. Study Procedure

Surveys were administered via a cross-sectional online design from 31 January 2024 to 13 March 2024.

The survey was administered in two sequential components. Participants first completed the WBI through the SurveyMonkey platform. Upon completion, participants were automatically redirected to the AMA Organizational Biopsy^®^ platform for the Mini-Z 2.0, a work–life and burnout instrument, as well as additional demographic and practice-related questions. The AMA Organizational Biopsy^®^ survey was accessible only through this redirect process and was not independently distributed or available through a separate survey link. CARRA facilitated distribution through its membership network, and the AMA provided the platform and aggregate reporting for this project. The organizations have no financial relationship, and collaboration was limited to this survey rather than an ongoing partnership.

Because the WBI and AMA Organizational Biopsy^®^/Mini-Z 2.0 datasets were collected on separate platforms without respondent-level linkage, workplace characteristics obtained from the AMA platform were analyzed descriptively and could not be evaluated as individual-level predictors of WBI outcomes. No imputation was performed. Analyses were conducted using completed survey instruments, and respondents were included only in analyses for which sufficient data were available. Because not all participants completed both survey components, denominators varied across analyses. All 164 respondents included in the WBI analyses had complete responses for the seven scored WBI items and the two WBI anchor items.

### 2.4. Statistical Analysis

Statistical analyses were performed using R version 4.4.1 (R Foundation for Statistical Computing, Vienna, Austria) using standard base-R statistical functions. Continuous variables were summarized using means and standard deviations (SDs) or medians and interquartile ranges (IQRs), as appropriate. Categorical variables were summarized using counts and percentages. Wilson score 95% confidence intervals (CIs) were calculated for proportions reported for WBI risk categories, burnout prevalence, and workplace measures. WBI scores were calculated as the sum of “Yes” responses to the seven scored dichotomous items (range 0–7); the two response-scaled anchor items were analyzed separately. Differences in mean WBI scores across observed anchor-item response categories were assessed using one-way analysis of variance (ANOVA), with eta-squared (η^2^) and Cohen’s f reported as measures of effect size. Assumptions of approximate normality of residuals and homogeneity of variance were evaluated using Shapiro–Wilk and Levene tests, respectively. To assess the robustness of findings, Welch’s ANOVA and Kruskal–Wallis tests were performed. For analyses involving ordered response categories, Spearman’s rank correlation was used to evaluate monotonic trends. A sensitivity analysis repeated the work–life integration analysis after excluding the WBI item “felt that all things you had to do were piling up so high that you could not overcome them”. This modified six-item score was exploratory and is not a validated WBI score.

Three exploratory post hoc subgroup comparisons were performed: burnout symptoms by gender, high job-related stress by gender, and burnout symptoms among respondents 6–10 versus >20 years after fellowship. Comparisons of proportions were evaluated using Fisher’s exact tests. Differences in proportions are presented with confidence intervals calculated using the Newcombe–Wilson method without continuity correction. Because these analyses were exploratory and were not prespecified in the statistical analysis plan, *p*-values were not adjusted for multiple comparisons and should be interpreted cautiously. A two-sided *p*-value < 0.05 was considered statistically significant. AMA Organizational Biopsy^®^/Mini-Z 2.0 data were available only in aggregate form; therefore, workplace measures could not be evaluated as individual-level predictors of WBI scores, and multivariable linkage analyses were not possible. Because all eligible CARRA-member pediatric rheumatologists identified through the membership directory were invited to participate, no a priori sample-size calculation was performed; the study represented an attempted census of the accessible eligible population.

## 3. Results

**Figure 1:**
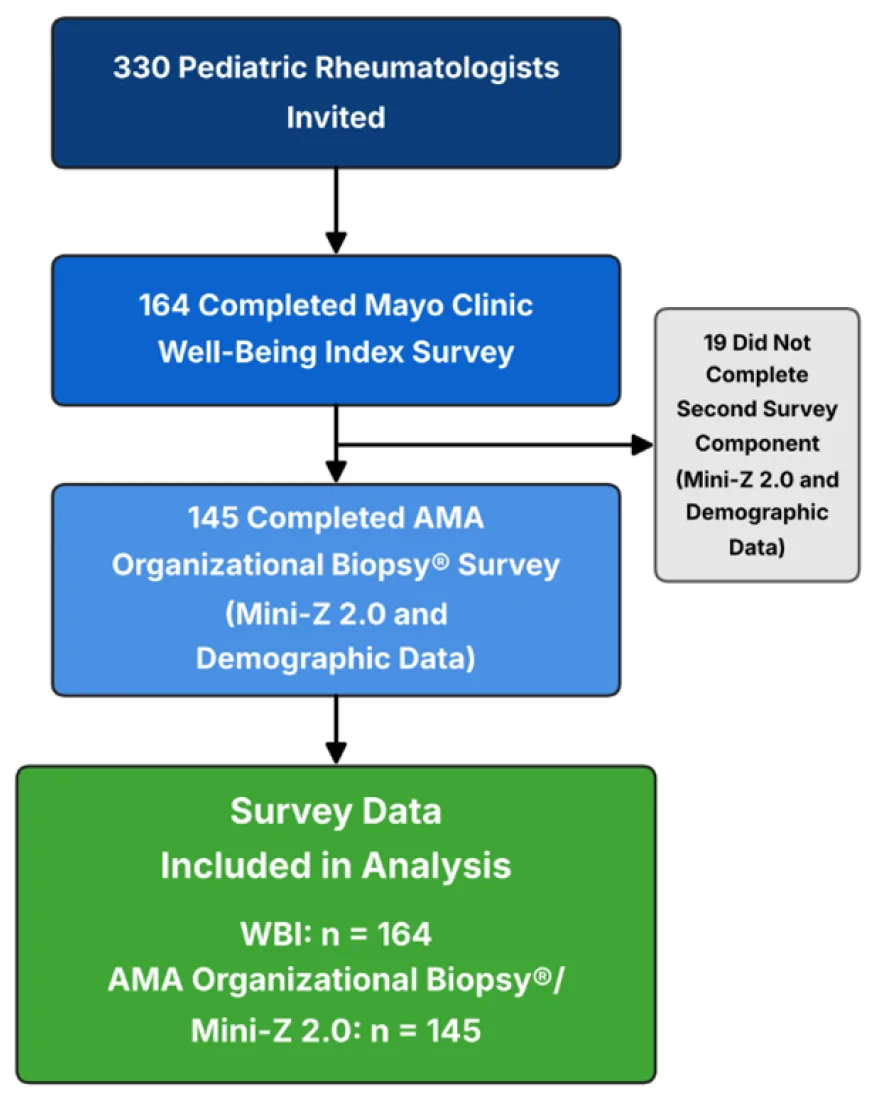
Participant recruitment and survey completion flow diagram.

A total of 330 pediatric rheumatologists who were registered CARRA members were invited to participate. Of these, 164 (49.7%) completed the WBI survey. Among the 164 respondents who completed the WBI, 145 (88.4%) subsequently completed the AMA Organizational Biopsy^®^ platform, which included the Mini-Z 2.0 survey and demographic questions; thus, 145 of the 330 invited physicians (43.9%) completed both survey components (Figure 1). Percentages are reported using the available denominator for each instrument or item.

### 3.1. Demographic and Practice Characteristics

Demographic and practice-related characteristics of respondents are summarized in Table 1. Of 145 respondents who completed the AMA Organizational Biopsy^®^ survey, the majority were female (73.1%). Respondents represented all major United States geographic regions, and 9.0% practiced in Canada. Most participants practice in university-based academic medical centers (69%) or teaching hospitals affiliated with medical school (24.1%). Years since fellowship completion were broadly distributed, with approximately one-quarter of respondents reporting 1 to 5 years (24.8%) and more than 20 years (24.8%). Nearly half of respondents practiced in small pediatric rheumatology centers (45.5%). Most physicians reported being on call for pediatric rheumatology 50% or less of the year (85.5%), and 63.4% reported institutional expectations to provide all in-person inpatient consultations during weekends. Additional practice-related information obtained through the AMA Organizational Biopsy^®^ platform included workplace culture characteristics, workload measures, and perceptions of organizational support (Table 1 and Table 2).

### 3.2. Workplace Culture and Organizational Factors

**Figure 2:**
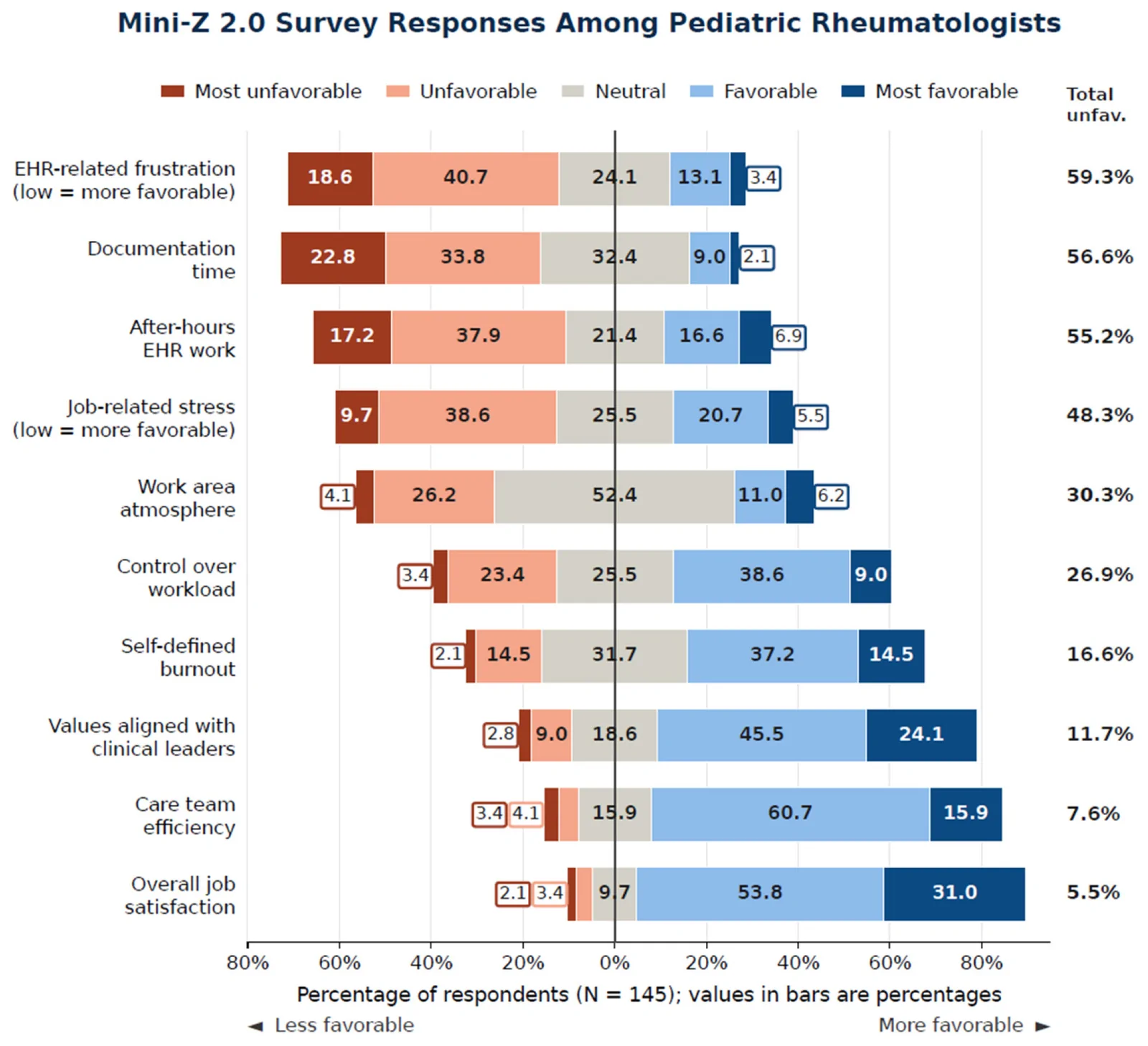
**Distribution of Mini-Z 2.0 survey responses among pediatric rheumatologists (N = 145).** Diverging stacked bar graph depicting the percentage distribution of responses across Mini-Z 2.0 survey domains among pediatric rheumatologists. Survey items assessed overall job satisfaction, self-defined burnout, alignment of values with clinical leaders, care-team efficiency, control over workload, work-related stress, documentation burden, EHR use outside working hours, EHR-related frustration, and workplace atmosphere. Responses are grouped into five categories ranging from most favorable to most unfavorable and are presented as percentages of survey respondents.

Responses from the Mini-Z 2.0 and AMA Organizational Biopsy^®^ surveys identified several workplace factors relevant to physician stress and professional well-being (Table 2). Many respondents reported positive perceptions of teamwork and found their clinical work to be meaningful. However, substantial proportions of physicians also identified workload burden, administrative responsibilities, and after-hours EHR-related tasks as important contributors to occupational stress. Nearly half of respondents (48.3%) reported high job-related stress, compared descriptively with 50.7% in the 2023 AMA National Physician Comparison Report [13]; the difference was 2.4 percentage points, although this comparison should be interpreted cautiously because the samples and benchmark methodology differ. Detailed workplace response distributions and confidence intervals are presented in Table 2 and Figure 2.

### 3.3. Burnout and Well-Being Outcomes

The mean WBI score was 2.7 (SD 1.9), with scores ranging from 0 to 7. Fifty-nine of 164 respondents (36.0%) had WBI scores ≥ 4, the validated threshold associated with increased risk of adverse physician outcomes [9]. The most frequently endorsed WBI items were feeling burned out from work (64.0%), feeling that work demands were overwhelming (59.1%), being bothered by emotional problems (48.8%), and worrying that work was hardening respondents emotionally (47.6%). The Mini-Z 2.0 single-item burnout measure indicated that 48.3% of respondents reported at least one symptom of burnout (Table 2). The 2023 AMA National Physician Comparison Report, based on responses from more than 12,400 physicians across 31 states and 81 health systems and organizations, reported 48.2% burnout and 50.7% high job-related stress [13]. This study estimate of 48.3% was similar descriptively to the AMA burnout benchmark, but this is not a formal equivalence test, and the samples, survey administration, and reporting methods may differ. The Mini-Z item asks respondents to choose a response based on their own definition of burnout, and includes ordered response options ranging from no symptoms to symptoms that will not go away or feeling completely burned out; the WBI item asks whether respondents felt burned out from work during the past month and uses a yes/no response. These measures therefore provide related but non-equivalent indicators and should not be treated as interchangeable. Most respondents (95.7%) agreed or strongly agreed that their work was meaningful (Table 3). Because one component of the WBI assesses being overwhelmed by workload (“felt that all things you had to do were piling up so high that you could not overcome them”), a sensitivity analysis was performed after removing this item from the WBI score. The association between work–life integration and well-being remained significant (F = 12.96, *p* < 0.001; η^2^ = 0.25). A significant monotonic trend across work–life integration categories also persisted (Spearman ρ = −0.50, *p* < 0.001), suggesting that the observed relationship was not driven solely by the workload-related WBI item.

**Figure 3:**
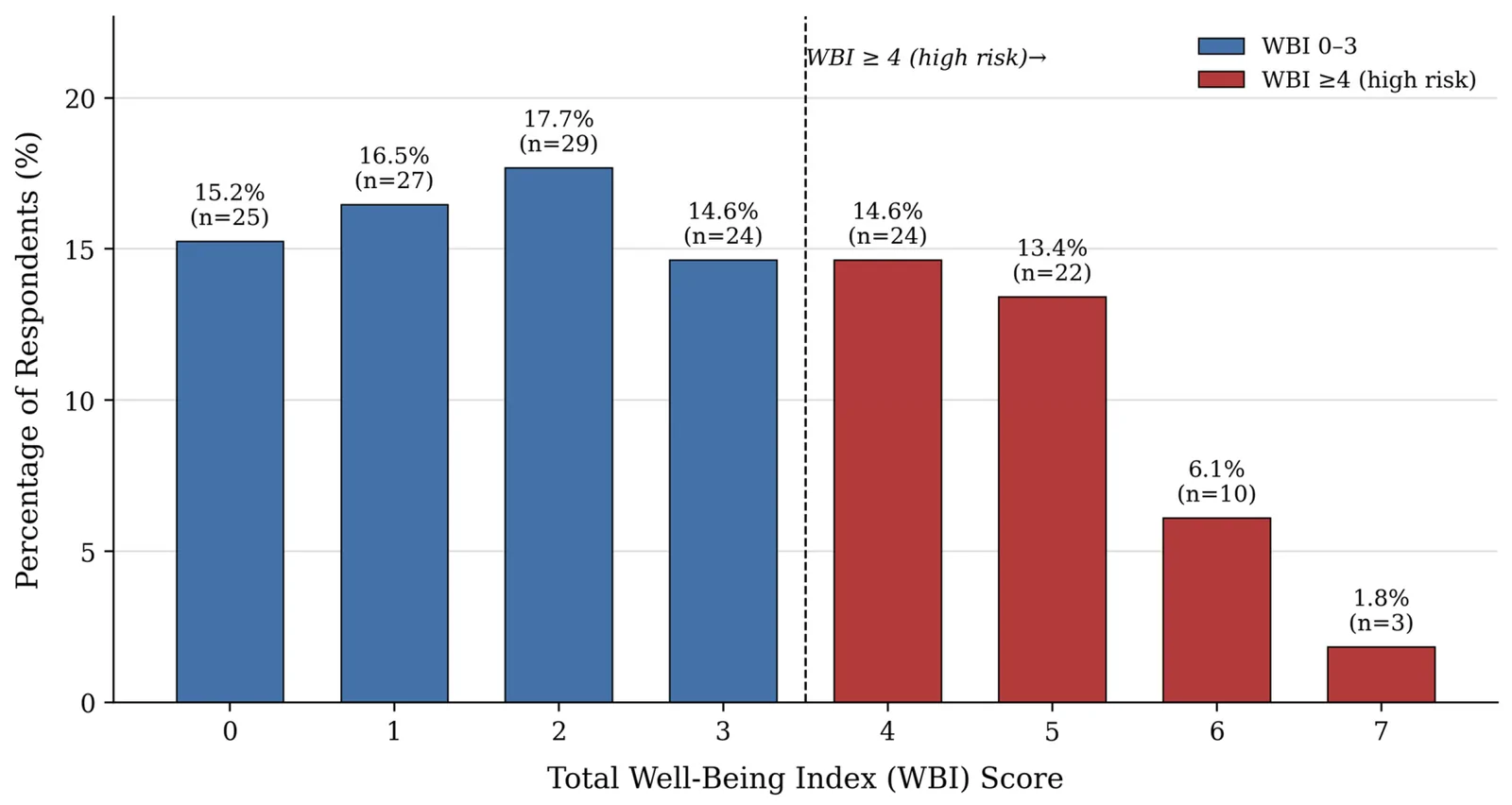
**Distribution of Mayo Clinic Well-Being Index total scores.** Note: Percentage of respondents (N = 164) by total Mayo Clinic Well-Being Index score. Scores range from 0 to 7 based on the sum of “Yes” responses across seven items. Scores ≥4 (shaded red) are associated with increased risk of adverse physician outcomes, including suicidal ideation and serious thoughts of leaving medicine.

Burnout and well-being outcomes are summarized in Table 4 and Figure 2 (Mini-Z 2.0), as well as Figure 3 (WBI).

**Table 3:** Mayo Clinic Well-Being Index (WBI) scores by work–life integration and meaningfulness of work response categories (N = 164).

Response Category	n (%)	Mean WBI Score (SD)	*p*-Value
***“The work I do is meaningful to me.”***			**0.26**
Agree strongly	101 (61.6)	2.5 (1.9)	
Agree	56 (34.1)	3.0 (2.0)	
Neither agree nor disagree	7 (4.3)	2.9 (2.3)	
Disagree	0 (0)	—	
Strongly disagree	0 (0)	—	
***“My work schedule leaves me enough time for my personal/family life.”***			**<0.001**
Agree strongly	15 (9.1)	0.7 (1.2)	
Agree	58 (35.4)	2.0 (1.7)	
Neither agree nor disagree	28 (17.1)	2.7 (1.8)	
Disagree	50 (30.5)	3.7 (1.6)	
Strongly disagree	13 (7.9)	4.4 (1.9)	

Note: WBI, Well-Being Index; SD, standard deviation. The WBI total score was calculated using the seven scored yes/no WBI items (range 0–7) and did not include the two response-scaled anchor items shown in this table. *p*-values are from one-way ANOVA comparing the mean WBI score across response categories with observed respondents; categories without respondents were excluded. Meaningful work: (2, 161) = 1.36, *p* = 0.26, η^2^ = 0.017. Work–life integration: F(4, 159) = 15.79, *p* < 0.001, η^2^ = 0.28. ANOVA assumptions were assessed using Shapiro–Wilk and Levene tests. Sensitivity analyses using Welch’s ANOVA and Kruskal–Wallis testing yielded similar results. For the work–life integration analysis, a significant monotonic trend across ordered response categories was observed (Spearman ρ = −0.536, *p* < 0.001). Sensitivity analysis excluding the WBI workload-related item (“felt that all things you had to do were piling up so high that you could not overcome them”) yielded similar results (F = 12.96, *p* < 0.001; η^2^ = 0.25).

**Table 4:** Burnout and Well-Being Index (WBI) outcomes (N = 164).

Outcome Variable	Value	95% CI
***Overall Well-Being Index (WBI) score***		
Mean WBI score (SD) ^1^	2.7 (1.9)	
Median WBI score (IQR) ^1^	3.0 (1.0–4.0)	
WBI score ≥ 4 (high risk) ^2^	59 (36.0)	29.0–43.6%
***Individual item endorsement***		
Burned out from work ^3^	105 (64.0)	56.4–71.0%
Work hardening emotionally ^4^	78 (47.6)	40.1–55.2%
Demands at work overwhelming ^5^	97 (59.1)	51.5–66.4%
Bothered by emotional problems ^6^	80 (48.8)	41.2–56.4%
Physical health interfering with work ^7^	32 (19.5)	14.2–26.2%
Feeling down, depressed, or hopeless ^8^	38 (23.2)	17.4–30.2%
Fell asleep while sitting inactive in public ^9^	14 (8.5)	5.2–13.8%

Note: WBI, Well-Being Index. Wilson score 95% CIs are reported for all item-level proportions. The WBI score is calculated from the seven yes/no items of the WBI (range 0–7). SD, standard deviation; IQR, interquartile range. ^1^ Mayo Clinic Well-Being Index total score, calculated from the seven items listed below. ^2^ WBI scores ≥4 are associated with increased risk of adverse physician outcomes, including suicidal ideation and serious thoughts of leaving medicine. ^3^ Item asked: “During the past month, have you felt burned out from your work?” ^4^ Item asked: “During the past month, have you worried that your work is hardening you emotionally?” ^5^ Item asked: “During the past month, have you felt that all things you had to do were piling up so high that you could not overcome them?” ^6^ Item asked: “During the past month, have you been bothered by emotional problems (such as feeling anxious, depressed, or irritable)?” ^7^ Item asked: “During the past month, has your physical health interfered with your ability to do your daily work at home and/or away from home?” ^8^ Item asked: “During the past month, have you often been bothered by feeling down, depressed, or hopeless?” ^9^ Item asked: “During the past month, have you fallen asleep while sitting inactive in a public place?”.

WBI scores differed significantly across levels of perceived work–life integration (one-way ANOVA F(4, 159) = 15.79, *p* < 0.001, η^2^ = 0.28, Cohen’s f = 0.63). Mean WBI scores increased progressively from 0.7 (SD 1.2) among respondents who strongly agreed that their work schedule allowed adequate time for personal and family life to 4.4 (SD 1.9) among those who strongly disagreed. A significant monotonic trend was observed across ordered response categories (Spearman ρ = −0.536, *p* < 0.001). Results were similar in the sensitivity analysis of work–life integration, which excluded the workload-related WBI item (“felt that all things you had to do were piling up so high that you could not overcome them”) (F(4, 159) = 12.96, *p* < 0.001, η^2^ = 0.246). In contrast, mean WBI scores did not differ significantly across categories of perceived work meaningfulness (F(2, 161) = 1.36, *p* = 0.26, η^2^ = 0.017). Mean WBI scores were 2.5 (SD 1.9), 3.0 (SD 2.0), and 2.9 (SD 2.3) among respondents who strongly agreed, agreed, or neither agreed nor disagreed that their work was meaningful, respectively. No respondents selected disagree or strongly disagree categories.

Exploratory comparisons based on AMA subgroup counts suggested differences in burnout symptoms and job-related stress by gender and career stage (Table 5). Burnout symptoms were reported by 56/106 (52.8%) female physicians compared with 13/38 (34.2%) male physicians (difference 18.6%, 95% CI 0.1–34.6%, *p* = 0.059). High job-related stress was reported by 57/106 (53.8%) female physicians compared with 12/38 (31.6%) male physicians (difference 22.2%, 95% CI 3.7–37.7%, *p* = 0.023). Burnout symptoms were reported by 19/30 (63.3%) physicians 6–10 years after fellowship compared with 11/36 (30.6%) physicians more than 20 years after fellowship (difference 32.8%, 95% CI 8.6–52.2%, *p* = 0.013). These comparisons were calculated from aggregate subgroup counts, were not adjusted for potential confounding, and should be considered exploratory. The subgroup data were not linked to individual WBI responses, so these comparisons do not establish associations between workplace measures and individual WBI scores.

**Figure 4:**
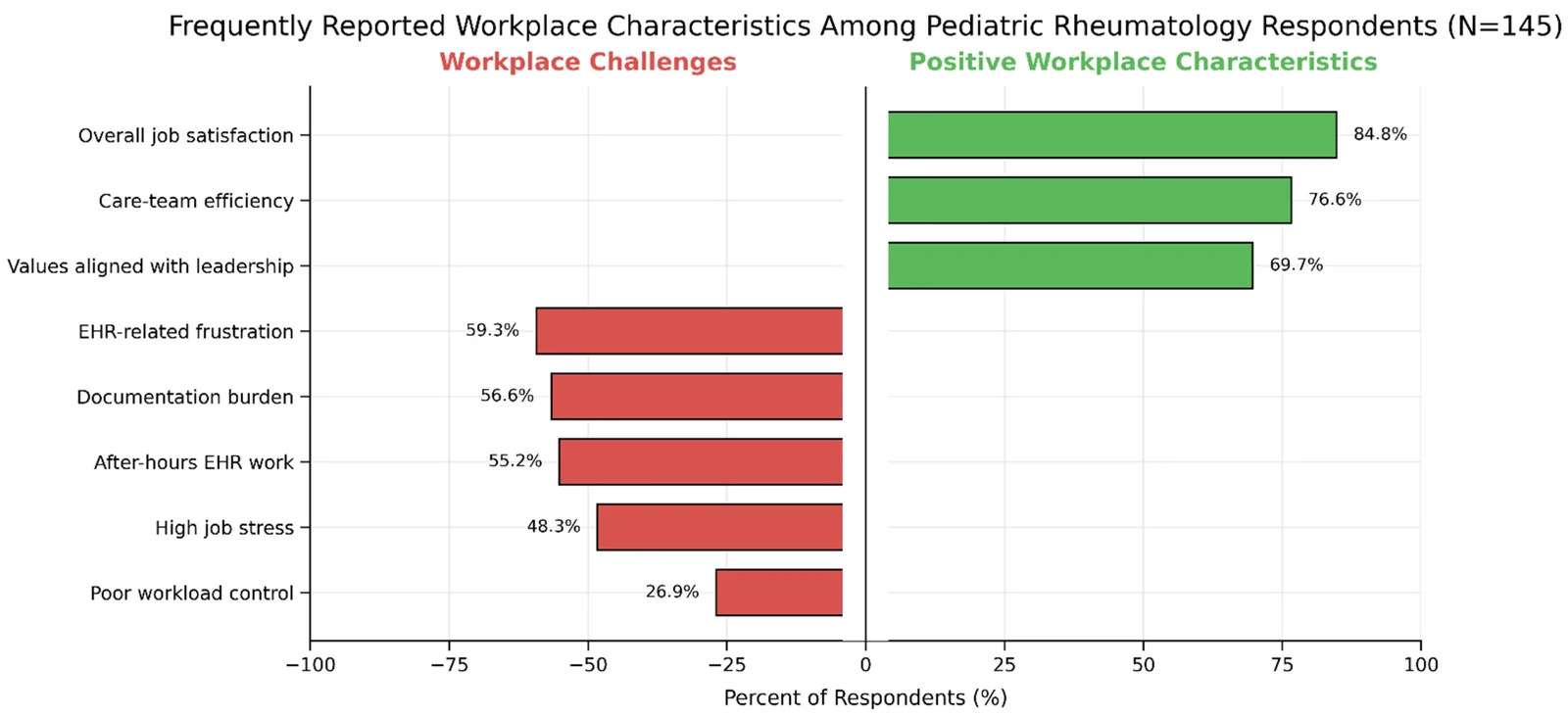
**Frequently reported workplace characteristics among pediatric rheumatology respondents (N = 145).** Red bars represent frequently reported workplace challenges. Green bars represent frequently reported positive workplace characteristics. Percentages indicate the proportion of pediatric rheumatologists endorsing each factor (N = 145). Percentages represent descriptive frequencies and do not indicate statistical associations with individual WBI scores because workplace and WBI datasets could not be linked at the respondent level.

Workplace stressors were commonly reported and included documentation burden, reduced work control, and after-hours EHR-related work. Positive workplace characteristics, including efficient teamwork and alignment of professional values with leadership, were also frequently reported. AMA Organizational Biopsy^®^/Mini-Z 2.0 data were available only in aggregate summaries. Therefore, workplace characteristics could not be linked to individual WBI scores or interpreted as individual-level predictors of distress. These descriptive frequencies are summarized in Table 2 and illustrated in Figure 4.

## 4. Discussion

To our knowledge, this study provides one of the first specialty-specific assessments of physician well-being, burnout symptoms, and workplace characteristics among North American pediatric rheumatologists using validated assessment instruments. This study additionally identified commonly reported workplace challenges that may represent targets for future organizational assessment and intervention. More than one-third of respondents had WBI scores associated with an increased risk of adverse professional and personal outcomes, while nearly one-half endorsed burnout symptoms on the Mini-Z 2.0 single-item measure. These findings provide specialty-specific descriptive data relevant to ongoing discussions about workforce well-being. Prior longitudinal research has linked physician burnout with subsequent reductions in professional work effort [14], but this study did not measure attrition, recruitment outcomes, changes in clinical effort, patient waiting times, or access to care. It also cannot establish that burnout in this cohort is higher than in other pediatric subspecialties or adult rheumatology.

The AMA Organizational Biopsy^®^ and Mini-Z 2.0 findings provide insight into workplace factors associated with physician well-being in pediatric rheumatology. Although most respondents reported positive care-team efficiency, nearly one-half reported substantial job-related stress, and more than one-half reported EHR-related frustration, documentation burden, and moderate-to-high levels of after-hours EHR work. Together, these findings suggest that workplace distress may coexist with positive perceptions of team functioning, indicating that strong teamwork alone may not fully mitigate the impact of administrative and documentation-related demands. These workplace challenges may represent important targets for future organizational assessment and intervention. Consistent with national frameworks, our findings support the view that clinician burnout is not solely an individual concern but also an organizational challenge that requires system-level approaches in addition to individual-focused strategies [2,15].

Exploratory post hoc analyses demonstrated higher rates of self-reported burnout symptoms and job stress among female pediatric rheumatologists and higher burnout symptom rates among physicians 6–10 years after training compared with physicians more than 20 years after training. Because these analyses were exploratory, unadjusted for multiple comparisons, and derived from aggregate subgroup summaries, the findings should be interpreted cautiously and considered hypothesis-generating. Future studies using respondent-level data are needed to determine whether these observed differences persist after adjustment for workload, family responsibilities, practice structure, and other organizational factors. The observed pattern of higher burnout and stress among female physicians is consistent with prior rheumatology workforce studies that have also identified gender differences in physician wellness and burnout [6].

A central finding was the coexistence of a strong sense of professional meaning and substantial occupational distress. More than 95% of respondents reported that their work was meaningful, yet 36.0% had WBI scores ≥4 and 48.3% reported burnout symptoms on the Mini-Z 2.0 single-item measure. These findings suggest that physicians may derive substantial meaning from their work while simultaneously experiencing significant occupational stress and burnout symptoms. Burnout estimates varied across assessment instruments, likely reflecting differences in item wording, scoring structure, recall periods, and measurement constructs. Accordingly, the Mini-Z burnout measure and the WBI burnout item should be interpreted as complementary rather than interchangeable indicators of physician distress. Together, these findings suggest that interventions focused solely on individual resilience may be insufficient without concurrent attention to organizational and workforce factors that contribute to physician well-being.

Work–life integration was strongly associated with WBI scores in this cross-sectional sample. WBI scores increased progressively as respondents reported having less time for their personal and family lives, with physicians who strongly disagreed that their work schedule allowed adequate personal or family time demonstrating mean scores above the validated threshold associated with an increased risk of adverse physician outcomes. The magnitude of the observed association was notable (η^2^ = 0.28). Importantly, this association remained significant in a sensitivity analysis which excluded the WBI item most directly related to workload (“felt that all things you had to do were piling up so high that you could not overcome them”), with only modest attenuation of the effect size (η^2^ decreased from 0.28 to 0.25). These findings suggest that the association between work–life integration and physician well-being is not solely attributable to overlap between these constructs. Nevertheless, the cross-sectional design and conceptual relationship between work–life integration and physician well-being preclude causal interpretation. Taken together, these findings suggest that work–life integration may represent an important area for future investigation and organizational well-being initiatives.

Documentation burden, after-hours EHR use, and administrative responsibilities were among the most frequently reported workplace challenges. These findings are consistent with prior studies describing documentation burden associated with EHR use [16] and burnout and wellness challenges among rheumatologists [6]. More than half of respondents rated time spent on documentation as marginal or poor, reported excessive after-hours EHR use, or indicated that the EHR contributed to workplace frustration. Because these factors were frequently reported by respondents, they may represent important areas for future organizational assessment and intervention. Potential strategies to address documentation burden include EHR workflow optimization, standardized documentation templates, medical scribes, and emerging ambient artificial intelligence-assisted documentation tools. Early studies suggest that digital and ambient AI scribes may reduce documentation time, cognitive burden, and, in some settings, physician burnout [17,18]. Additional interventions, including protected academic time, flexible scheduling, telehealth visits, adequate clinical and administrative staffing, and expanded team-based care models, may further improve work–life integration while preserving clinical productivity.

Although burnout symptoms and occupational distress were common in this sample, direct comparisons with other pediatric subspecialties and adult rheumatology populations should be interpreted cautiously because differences in survey instruments, sampling strategies, and time periods may affect estimates. Nevertheless, occupational distress has also been reported in broader rheumatology populations. For example, the 2020 Canadian Rheumatology Association Workforce and Wellness Survey identified substantial concerns related to physician wellness and burnout among practicing rheumatologists [6]. Cross-study comparisons should be interpreted cautiously because different instruments and methodologies were used.

Pediatric rheumatology continues to face substantial workforce shortages, with an inadequate number of physicians to meet current and projected demand [4,5]. In other physician populations, longitudinal evidence has linked burnout and declining professional satisfaction to subsequent reductions in professional work effort [14]. Although these consequences are relevant to a specialty facing workforce shortages, they were not measured in the present study and cannot be inferred directly from our findings. Recent data demonstrating substantial burnout among pediatric rheumatology fellows despite generally high levels of resilience suggest that concerns regarding physician well-being may emerge early in training and span the career continuum [7]. Future longitudinal studies following pediatric rheumatologists from fellowship through independent practice could help characterize trajectories of well-being and burnout over time and identify critical periods for intervention.

Compensation was not assessed in this study and therefore cannot be evaluated as a contributor to physician well-being or burnout in this cohort. Nevertheless, workforce reports have identified compensation differences between pediatric and adult rheumatology, highlighting financial considerations as another potential workforce issue that warrants further study in pediatric rheumatology [19]. Future studies should directly evaluate the relationship between compensation, workload, professional satisfaction, and physician well-being within pediatric rheumatology. Despite substantial occupational distress, respondents reported high levels of work meaningfulness. This finding suggests that professional meaning and occupational distress are not mutually exclusive and reinforces the importance of addressing organizational contributors to well-being even among physicians who derive substantial satisfaction from their work. Organizational strategies targeting workflow inefficiencies, administrative burden, staffing support, and work–life integration may represent promising areas for future investigation. Collectively, these findings suggest that pediatric rheumatologists may experience substantial occupational distress despite reporting a strong sense of professional meaning. Addressing potentially modifiable organizational and workflow-related factors may represent an important component of efforts to support physician well-being and workforce sustainability. Continued longitudinal assessment of physician well-being will be essential to evaluate the effectiveness of organizational interventions, monitor workforce trends, and inform future healthcare policy initiatives. The AMA Organizational Biopsy^®^ platform allows for repeated assessments and benchmarking over time, providing a valuable tool for the pediatric rheumatology community to consider tracking workforce well-being and evaluation of organizational interventions longitudinally.

## 5. Limitations

This study has several limitations. First, participation was voluntary and limited to CARRA-member pediatric rheumatologists, creating the potential for selection bias and nonresponse bias. Because demographic and practice information was unavailable for nonrespondents, respondent–nonrespondent comparisons could not be performed. Consequently, the direction and magnitude of potential nonresponse bias cannot be determined. Because physicians experiencing greater occupational distress may have been more motivated to participate or, alternatively, may have been less likely to participate because of time constraints or survey fatigue, the observed prevalence estimates may not fully represent the broader CARRA-member pediatric rheumatology workforce. Although respondents represented diverse practice settings, career stages, and geographic regions across North America, the majority practiced in academic medical centers and were based in the United States, which may limit generalizability to all pediatric rheumatologists. Although direct comparisons between respondents and nonrespondents were not possible, the demographic composition of respondents was generally consistent with published pediatric rheumatology workforce data. Women comprised 73.1% of survey respondents, similar to the 71% female representation reported among currently board-certified pediatric rheumatologists in the United States [4]. These findings provide some support for the representativeness of the sample with respect to gender distribution, although representativeness across other characteristics could not be assessed.

Second, the cross-sectional design precludes conclusions regarding causality or temporal relationships. The WBI was administered before the AMA Organizational Biopsy^®^/Mini-Z 2.0 component. Although this sequence was applied consistently to all participants, the possibility of survey-order effects cannot be excluded, because completion of well-being and burnout items may have influenced responses to subsequent workplace culture and organizational questions. The study design did not permit direct evaluation of the magnitude or direction of any such effect.

The WBI and AMA Organizational Biopsy^®^/Mini-Z 2.0 instruments were administered through separate platforms and could not be linked at the individual respondent level. As a result, workplace characteristics could only be analyzed descriptively, and direct associations between organizational factors and individual well-being outcomes could not be evaluated. Furthermore, AMA Organizational Biopsy^®^ data were available only in aggregate form, limiting opportunities for respondent-level analyses and multivariable modeling. Although exploratory subgroup comparisons were performed, these analyses were post hoc and limited to aggregate subgroup data. Findings should therefore be considered as hypothesis-generating and interpreted cautiously. Differences in instrument structure, recall periods, scoring approaches, and operational definitions of burnout may also contribute to variations in observed burnout estimates across measures.

Third, all measures relied on self-reported survey data and therefore may be subject to recall bias, response bias, and social desirability bias. Data collection occurred during January–March 2024 and may have been influenced by temporal or seasonal factors not captured in the survey. Additionally, compensation, institutional resources, staffing levels, and other contextual workplace variables were not collected and may influence physician well-being and burnout.

Finally, the strong association observed between work–life integration and WBI score should be interpreted with caution because the work–life integration anchor item is conceptually related to several domains of occupational distress measured by the WBI. Although this item was excluded from calculation of the seven-item WBI score, conceptual overlap between work–life integration and physician well-being remains possible. However, the observed association persisted in a sensitivity analysis excluding the workload-related WBI item most closely related to work–life integration, suggesting that the finding was not solely attributable to overlap between constructs. Nevertheless, this association does not establish that an independently measured organizational exposure causes physician distress. Despite these limitations, this study provides a substantial recent assessment of physician well-being, burnout symptoms, and workplace characteristics among pediatric rheumatologists and offers descriptive data that may inform future investigations and organizational well-being initiatives.

## 6. Conclusions

Burnout symptoms and occupational distress were common among responding North American pediatric rheumatologists, despite high reported levels of work meaningfulness. Frequently reported workplace challenges included documentation burden, EHR-related frustration, job-related stress, and limited work–life integration. Although WBI scores differed across response categories for the work–life integration anchor item, the cross-sectional design precludes causal conclusions, and workplace characteristics from the AMA Organizational Biopsy^®^ assessment could not be linked to individual WBI responses. These findings provide specialty-specific descriptive data to inform future research and organizational efforts to support physician well-being in pediatric rheumatology. Longitudinal and respondent-level studies are needed to further evaluate factors associated with occupational distress and to assess the effectiveness of organizational interventions aimed at supporting physician well-being and workforce sustainability within pediatric rheumatology.

## Figures and Tables

**Table 1:** Demographic and practice characteristics of responding CARRA-member pediatric rheumatologists who completed the AMA Organizational Biopsy^®^ survey (N = 145).

Characteristic	N (%)
Total respondents	145
***Gender identity***	
Female *	106 (73.1)
Male	38 (26.2)
Transgender	1 (0.7)
Prefer not to answer	0 (0)
***Race/ethnicity*** ^1^	
White *	106 (73.1)
Asian	26 (17.9)
Black or African American	3 (2.1)
Latinx/Latino/Latina or Hispanic	5 (3.4)
Middle Eastern or North African	4 (2.8)
American Indian or Alaska Native	1 (0.7)
Native Hawaiian or Pacific Islander	0 (0)
Other ^2^	1 (0.7)
Prefer not to answer	7 (4.8)
***Geographic location of practice institution***	
Northeast	36 (24.8)
Midwest	32 (22.1)
Southeast	23 (15.9)
Southcentral	13 (9.0)
Mountain West	7 (4.8)
Pacific West	17 (11.7)
Canada	13 (9.0)
Prefer not to answer	4 (2.8)
***Years since fellowship completion***	
1–5 years	36 (24.8)
6–10 years	30 (20.7)
11–15 years	23 (15.9)
16–20 years	20 (13.8)
>20 years	36 (24.8)
***Practice setting***	
University-based academic medical center	100 (69.0)
Teaching hospital with medical school affiliation	35 (24.1)
Teaching hospital with no medical school affiliation	3 (2.1)
Non-academic community hospital	2 (1.4)
Multi-specialty physician group	2 (1.4)
Private practice	1 (0.7)
Locums	0 (0)
Other ^3^	2 (1.4)
***Practice size*** ^4^	
Small center	66 (45.5)
Large center	79 (54.5)
***On-call frequency (% of year)*** ^5^	
0–25%	78 (53.8)
26–50%	46 (31.7)
51–75%	8 (5.5)
76–100%	11 (7.6)
Prefer not to answer	2 (1.4)
***Institutional expectation for weekend in-person consultation*** ^6^	
All inpatient consults	92 (63.4)
Urgent ICU-level consults only	5 (3.4)
Physician discretion	47 (32.4)
Telephone/telemedicine	1 (0.7)

Note: Percentages are based on the number of respondents with available data for each variable and may not sum to 100% due to rounding. * Additional review confirmed that the identical values reported for female gender and White race/ethnicity reflect the underlying dataset. ^1^ Respondents could select more than one race/ethnicity category; percentages sum to more than 100%. ^2^ Write-in response: “combination”. ^3^ Write-in responses: “government” and “US government research hospital”. ^4^ Small centers: ≤3 clinical full-time-equivalent (FTE) pediatric rheumatology physicians; large center: ≥4 clinical FTE pediatric rheumatology physicians. ^5^ Item asked: “What percentage of the year are you on call for pediatric rheumatology?” ^6^ Item asked: “What is your institution’s expectation to go into the hospital for consultations on weekends?”.

**Table 2:** Workplace culture and organizational factors (N = 145).

Workplace Variable	N (%)	95% CI
***EHR and documentation burden***		
Time spent on documentation rated marginal or poor ^1^	82 (56.6)	48.5–64.3%
After-hours EHR time rated moderately high or excessive ^2^	80 (55.2)	47.1–63.0%
EHR adds to daily frustration (agree/strongly agree) ^3^	86 (59.3)	51.2–67.0%
***Workload and stress***		
Control over workload rated marginal or poor ^4^	39 (26.9)	20.3–34.6%
High job-related stress (agree/strongly agree) ^5,^*	70 (48.3)	40.3–56.3%
***Team and organizational culture***		
Care-team efficiency rated good/optimal ^6^	111 (76.6)	69.0–82.7%
Professional values aligned with clinical leadership (agree/strongly agree) ^7^	101 (69.7)	61.8–76.6%
***Workplace atmosphere***		
Calm ^10^	9 (6.2)	3.3–11.4%
Occasionally busy ^10^	16 (11.0)	6.9–17.2%
Busy, but reasonable ^10^	76 (52.4)	44.3–60.4%
Very busy ^10^	38 (26.2)	19.7–33.9%
Hectic, chaotic ^10^	6 (4.1)	1.9–8.7%
***Satisfaction and burnout***		
Overall job satisfaction (agree/strongly agree) ^8^	123 (84.8)	78.1–89.8%
Burnout symptoms present ^9,^*	70 (48.3)	40.3–56.3%

Note: Workplace culture variables were obtained from aggregate Mini-Z 2.0 and AMA Organizational Biopsy^®^ survey data. The 95% CIs are Wilson score intervals. Unless noted otherwise, “positive/negative” response categories reflect the top or bottom two categories of a 5-point scale, combined. Abbreviations: EHR, electronic health record. ^1^ Item asked: “The amount of time I spend on documentation is…” (5-point scale: optimal, good, satisfactory, marginal, poor). “Marginal or poor” reported. ^2^ Item asked: “The amount of time I spend on the electronic health record (EHR) outside normal/scheduled work hours, including work done at home, is…” (5-point scale: minimal/none, modest, satisfactory, moderately high, excessive). “Moderately high or excessive” reported. ^3^ Item asked: “The electronic health record (EHR) adds to the frustration of my day”. “Agree or strongly agree” reported. ^4^ Item asked: “My control over my workload is…” (5-point scale: optimal, good, satisfactory, marginal, poor). “Marginal or poor” reported. ^5^ Item asked: “I feel a great deal of stress because of my job”. “Agree or strongly agree” reported. ^6^ Item asked: “The degree to which my care team works efficiently together is…” (5-point scale: optimal, good, satisfactory, marginal, poor). “Good or optimal” reported. ^7^ Item asked: “My professional values are well aligned with those of my clinical leaders”. “Agree or strongly agree” reported. ^8^ Item asked: “Overall, I am satisfied with my current job”. “Agree or strongly agree” reported. ^9^ Assessed via the single-item measure: “Using your own definition of ‘burnout,’ please choose one of the answers below”. “Burnout symptoms present” combines the three categories indicating at least one symptom of burnout (beginning to burn out, symptoms that will not go away, or feeling completely burned out), consistent with the standard single-item burnout measure validated against the Maslach Burnout Inventory cutoff [12]. * Additional review confirmed that the identical values reported for high job-related stress and burnout symptoms reflect the underlying dataset. ^10^ Item asked: “Which selection best describes the atmosphere in your primary work area?” Response categories are reported individually.

**Table 5:** Exploratory post hoc subgroup comparisons of burnout symptoms and job stress.

Comparison	Group 1, n/N (%)	Group 2, n/N (%)	Difference, %	95% CI	*p*-Value
Burnout symptoms by gender	Female 56/106 (52.8)	Male 13/38 (34.2)	18.6%	0.1–34.6%	0.059
High job stress by gender	Female 57/106 (53.8)	Male 12/38 (31.6)	22.2%	3.7–37.7%	0.023
Burnout symptoms by years post-fellowship	6–10 years 19/30 (63.3)	>20 years 11/36 (30.6)	32.8%	8.6–52.2%	0.013

Note: Burnout symptoms were defined as respondents selecting one of the following AMA Organizational Biopsy categories: (1) “I feel completely burned out. I am at the point where I may need to seek help”, (2) “The symptoms of burnout that I’m experiencing won’t go away. I think about work frustrations a lot”, or (3) “I am beginning to burn out and have one or more symptoms of burnout, e.g., emotional exhaustion”. High job stress was defined as responding “Agree” or “Agree strongly” to the statement “I feel a great deal of stress because of my job”. Differences in proportions are reported with Newcombe–Wilson 95% confidence intervals. *p*-values were calculated using two-sided Fisher exact tests. Analyses were exploratory, post hoc, and were not adjusted for multiple comparisons; therefore, results should be interpreted cautiously.

## Data Availability

The datasets generated and analyzed during the current study are not publicly available because the sample was drawn from a relatively small physician workforce and public release may increase the risk of participant re-identification. De-identified data may be made available from the corresponding author upon reasonable request and subject to institutional review and applicable confidentiality protections. The Mayo Clinic Well-Being Index and AMA Organizational Biopsy^®^ instruments are proprietary and therefore cannot be publicly distributed as part of the study dataset.
